# Noninvasive methods of diagnosing bladder outlet obstruction

**DOI:** 10.4103/0970-1591.45543

**Published:** 2009

**Authors:** Robert Pickard, Clive Griffiths

**Affiliations:** School of Surgical and Reproductive Sciences, Newcastle University, Newcastle upon Tyne, UK; 1 Department of Medical Physics, Freeman Hospital, Newcastle upon Tyne, UK

## WHY DO WE NEED CLINICAL MEASUREMENTS?

There are three main ways in which clinical measurements are used

1. To enhance accuracy of clinical assessment (diagnosis)
2. To facilitate clinical discussion with patients (communication)
3. To predict the likely outcome of treatment (prognosis)

## HOW ARE NEW CLINICAL MEASUREMENTS ASSESSED?

The development of novel clinical measurement techniques requires progression through a number of key stages. The first requirement is that there is a clinical need for the measurement to be performed and that the results will improve patient care. Next, readings obtained by the device have to be shown to be valid – that is they measure what they are supposed to measure; they have to be demonstrated to be reliable – that is the same result is obtained on repeat testing and also it has to be established that the new test is generalizable to the range of healthcare settings in which it is likely to be used. Finally, the results of the test have to be shown to make a difference to the diagnosis, management or treatment of the relevant clinical problem in a way that is an advance on existing care pathways and ‘gold standard’ tests. These requirements have recently been formalized in a consensus guideline.[1] Once all this has been established, purchasers of healthcare will generally require some independent assessment of the worth of the new technique in comparison to existing options prior to its introduction into routine practice.[2]

## WHAT IS THE TECHNOLOGY GAP IN DIAGNOSIS OF BLADDER OUTLET OBSTRUCTION?

The majority of (but not all) urologists would agree that measurement of voiding bladder pressure and urinary flow rate in order to categorize bladder outlet obstruction (BOO) in men complaining of bothersome lower urinary symptoms (LUTS) is a desirable aim. The reason is that it provides a pathophysiological basis for the discussion of treatment options and that a positive diagnosis of BOO increases the chance of a good outcome from the various forms of prostatectomy designed to correct obstruction.[3] The problem is that conventional bladder pressure measurement requires catheterization of the bladder in order to perform voiding cystometry otherwise known as a pressure-flow study (PFS). The relatively high resource use, restriction to specialized hospital departments and the risk of infection means that invasive PFS are not generalizable across the range of settings where they might be needed and hence are not widely used in the assessment of men with LUTS.[4] In this regard a noninvasive simpler method of categorizing BOO would be extremely useful for clinicians charged with assessing men with LUTS.

## WHAT ARE THE TRADITIONAL NONINVASIVE OPTIONS?

Much work has gone into defining whether existing clinical measures such as maximum flow rate (Q_max_), symptom score, prostate size or prostate specific antigen (PSA) may be useful in predicting the presence of BOO.[5] Although somewhat useful none of these measures has sufficient diagnostic accuracy to rival the ‘gold standard’ of invasive PFS and this probably reflects the indirect nature of the readings obtained with reference to BOO. A number of groups have therefore worked hard to develop new technologies that could potentially fulfill the clinical need and we are very fortunate in having them review their work and those of other clinical scientists in this symposium.

## WHAT ARE THE NEW DEVELOPMENTS IN NONINVASIVE DIAGNOSIS OF BLADDER OUTLET OBSTRUCTION?

The novel technologies basically consist of two types

1. Those that provide traditional urodynamic measurement in a noninvasive manner.
  - Direct measurement of isovolumetric bladder pressure - condom catheter
  - Indirect measurement of isovolumetric bladder pressure - penile cuff
  - Measurement of outlet resistance - Doppler ultrasound
2. Those that measure an alternative variable that may relate to BOO
  - Bladder wall weight
  - Perineal noise recording

The Authors of the following reviews have done a great job to summarize the evidence regarding their technique in a concise yet accessible way and we hope that they provide sufficient information for you as readers to decide which technology may be of potential use in your own clinical practice.
